# Mice Expressing a "Hyper-Sensitive" Form of the Cannabinoid Receptor 1 (CB_1_) Are Neither Obese Nor Diabetic

**DOI:** 10.1371/journal.pone.0160462

**Published:** 2016-08-08

**Authors:** David J. Marcus, Michael L. Zee, Brian J. Davis, Chris P. Haskins, Mary-Jeanette Andrews, Randa Amin, Angela N. Henderson-Redmond, Ken Mackie, Traci A. Czyzyk, Daniel J. Morgan

**Affiliations:** 1 Gill Center for Biomolecular Science, Indiana University, Bloomington, Indiana, 47405,United States of America; 2 Department of Psychological and Brain Sciences, Indiana University, Bloomington, Indiana, 47405, United States of America; 3 Department of Anesthesiology, Penn State College of Medicine, Hershey, Pennsylvania, 17033, United States of America; 4 Center for the Integrative Study of Animal Behavior, Indiana University, Bloomington, Indiana, 47405, United States of America; "INSERM", FRANCE

## Abstract

Multiple lines of evidence implicate the endocannabinoid signaling system in the modulation of metabolic disease. Genetic or pharmacological inactivation of CB_1_ in rodents leads to reduced body weight, resistance to diet-induced obesity, decreased intake of highly palatable food, and increased energy expenditure. Cannabinoid agonists stimulate feeding in rodents and increased levels of endocannabinoids can disrupt lipid metabolism. Therefore, the hypothesis that sustained endocannabinoid signaling can lead to obesity and diabetes was examined in this study using S426A/S430A mutant mice expressing a desensitization-resistant CB_1_ receptor. These mice display exaggerated and prolonged responses to acute administration of phytocannabinoids, synthetic cannabinoids, and endocannabinoids. As a consequence these mice represent a novel model for determining the effect of enhanced endocannabinoid signaling on metabolic disease. S426A/S430A mutants consumed equivalent amounts of both high fat (45%) and low fat (10%) chow control diet compared to wild-type littermate controls. S426A/S430A mutants and wild-type mice fed either high or low fat control diet displayed similar fasting blood glucose levels and normal glucose clearance following a 2 g/kg glucose challenge. Furthermore, S426A/S430A mutants and wild-type mice consumed similar amounts of chow following an overnight fast. While both THC and JZL195 significantly increased food intake two hours after injection, this increase was similar between the S426A/S430A mutant and wildtype control mice Our results indicate that S426A/S430A mutant mice expressing the desensitization-resistant form of CB_1_ do not exhibit differences in body weight, food intake, glucose homeostasis, or re-feeding following a fast.

## Introduction

The consequences of dysregulated metabolism, including obesity and diabetes, are among the most devastating health crises facing the developed world today. In the United States, diabetes mellitus is the 7^th^ leading cause of death, with more than 73,282 deaths in 2011 from the disease [1]. In addition, the prevalence of obesity among adults has increased from 12% in 1990 to 35.6% in 2010 [2]. The CDC estimated the annual cost of obesity in 2008 at $147 billion [3].

One intriguing therapeutic target for the treatment of metabolic disease is the endocannabinoid system (ECS). In the central nervous system, the endocannabinoid signaling system is comprised of the cannabinoid receptor 1 (CB_1_), its two primary endogenous ligands, N-arachidonoylethanolamide (also referred to as anandamide, AEA) and 2-arachidonoyl glycerol (2-AG), and also the biosynthetic and hydrolytic enzymes that synthesize and degrade endocannabinoids. Genetic and pharmacological manipulation has established the endocannabinoid system as a major player in metabolic regulation, ranging from the control of food intake to the regulation of adipogenesis, fatty acid oxidation and energy expenditure. Mice lacking CB_1_ are resistant to diet-induced obesity, are lean on a regular chow diet, and exhibit reduced food intake [4,5]. CB_1_ knock-out (KO) mice do not develop diet-induced insulin resistance [4]. In mice lacking CB_1_, food intake is decreased while energy expenditure is increased suggesting that both aspects could be involved in the leanness observed in CB_1_-deficient mutant mice [5]. Pharmacological blockade with a selective CB_1_ inverse agonist, rimonabant (SR141716), has been widely studied in rodents and humans as a potential treatment for obesity. Treatment of diet-induced obese rodents with daily rimonabant causes a transient reduction in food intake, with sustained decreases in body weight, adiposity, and fasting glycemia [6,7]. Rimonabant also prevents hepatic steatosis, a phenomenon where excess fat storage in the liver leads to cirrhosis [8]. Several studies have suggested that endocannabinoid modulation of the peripheral elements of metabolic homeostasis such as energy expenditure might be responsible for the weight-reducing effects of rimonabant [9–11].

Conversely, treatment with low doses of Δ^9^-THC increases the consumption and preference for highly-palatable foods [12–14]. Several studies in mice have demonstrated endocannabinoids are increased in diet-induced obesity (DIO). Higher levels of 2-AG have been reported in the lateral hypothalamus and in visceral adipose tissue of DIO mice [15–17]. Similar to mice, visceral fat collected from obese patients had higher levels of 2-AG [15]. Blocking 2-AG synthesis with a DAG lipase inhibitor acutely decreased high-fat diet (HFD) intake in mice [18]. Additionally, acute increases in endocannabinoid levels impair clearance of plasma triglycerides by apolipoprotein E [19]. Collectively, these findings suggest that an overactive endocannabinoid system may be obesogenic by influencing food choice and consumption, altering energy expenditure, and disrupting lipid metabolism. Furthermore, these effects may be exacerbated with exposure to high-fat diets.

In the present study, we have examined the effects of “chronically” enhanced endocannabinoid signaling on body weight regulation, food intake, and glucose homeostasis using S426A/S430A mutant mice. The S426A/S430A mutants express a knock-in mutation of serines 426 and 430 on the C-terminus of the CB_1_ receptor to alanines. These residues are putative G protein-coupled receptor kinase (GRK) targets and are required for β-arrestin2-mediated desensitization of CB_1_. Expression of these mutations blocked desensitization of CB_1_ in oocytes, transfected cells, and in mice [20–24]. In mice, this mutation results in increased acute responses to endocannabinoids, phytocannabinoids, and synthetic cannabinoids making these mice a novel model for studying the effects of “over-active” endocannabinoid signaling *in vivo* [24].

## Methods

### Animals

All animal care and experimental procedures used in this study were approved by the Institutional Animal Care and Use Committees of Indiana University Bloomington and Penn State University College of Medicine and conform to the Guidelines of the National Institutes of Health on the Care and Use of Animals. Mice were maintained on a 12 hour light dark cycle with lights on at 7AM and lights off at 7PM.

### Food Intake and Body Weight

Separate cohorts of male S426A/S430A and wild-type littermate mice were placed on either a high fat diet (HFD) (D12451, 45% kcal from fat) from Research Diets Inc. (New Brunswick, NJ), or a low fat control diet (LFD) (10% kcal from fat), at weaning. Body weight was measured weekly and food intake was measured every 1–3 days from weaning until 20 weeks of age. Food intake was calculated as the number of grams of food consumed per kilogram of body weight per day.

### Glucose Tolerance Test

Fasting glucose levels were measured in S426A/S430A mutant and wild-type littermate control mice that were given either HFD or LFD from weaning until 20 weeks of age. Glucose tolerance testing was done at 20 weeks of age. Prior to testing, mice were fasted overnight (5:00 PM-11:00 AM) for 18 hours. Fasting glucose levels were measured and then mice were given an intraperitoneal (IP) injection of 2g/kg D-glucose (Fisher Scientific, St. Louis, MO). Blood glucose was measured from blood collected via lateral tail vein nick just prior to injection (i.e. fasting blood glucose levels), and 15, 30, 60, and 120 minutes following glucose injection. Blood glucose levels were measured using a glucometer and Comfort Curve blood glucose test strips from Accu-Chek (Roche Diagnostics, Indianapolis, IN).

### Insulin Tolerance Test

Insulin tolerance was examined in 30 week old wild-type and S426A/S430A mutant littermate mice given either HFD or LFD from weaning until 30 weeks of age. Testing was done in mice that were fasted overnight for 18 hours (5:00 PM-11:00 AM). After fasting, mice received an IP injection of 1U/kg of insulin (Eli Lilly, Indianapolis, IN). Blood was collected via lateral tail vein nick prior to, and 15, 30, 60, and 120 minutes following Insulin injection. Blood glucose levels were assessed using a glucometer and blood glucose test strips as described for glucose tolerance experiments.

### Re-feeding Following an Overnight Fast

Re-feeding following an 18 hour overnight fast (5:00 PM-11:00 AM) was measured in 25 week old S426A/S430A mutant and wild-type littermate control mice fed HFD or LFD starting at weaning. Access to HFD or LFD was restored and food consumption was measured at 1, 4, 6 and 24 hours following resumption of food access.

### Δ^9^-THC-induced Feeding Dose Response Curve

Individually housed mice were habituated to HFD for one week prior to testing. Daily food intake and body weight measurements were recorded to establish a stable HFD consumption. Food consumption was calculated and normalized to body weight as grams of food per kilogram for each mouse. A dose response curve for Δ^9^-THC-induced feeding was done using a Latin-square design in which a complete dose-response curve was generated during each testing session across multiple mice (e.g. mice were divided into four groups A thru D and each group was administered a different IP Δ^9^-THC dose; 0,1,3 or 10mg/kg). Multiple testing sessions that were spaced at least 72 hours apart were performed to generate a complete dose response for each mouse. Food was removed one hour before onset of the dark cycle and *Δ*^*9*^-THC injections were given immediately prior to the dark cycle. Thirty minutes post-injection, five pellets of HFD were weighed and placed in each cage. Food pellets were weighed 1, 2 and 24 hours after HFD was given. Δ^9^-THC was dissolved in an 18:1:1 saline:Cremophor:ethanol vehicle.

### JZL195-induced Feeding

Mice were habituated to HFD, and food intake and body weight measurements were taken daily as described above for the Δ^9^-THC-induced feeding experiments. In this cohort, body composition was determined using TD-NMR (Bruker minispec LF90II) prior to, and after, 14 days of once-daily (IP) injections of 8mg/kg JZL195. Food consumption and body weights were measured daily. For each test day, HFD was removed one hour before the onset of the dark cycle and mice were injected with JZL195 immediately before the start of the dark cycle. Five pellets of HFD were pre-weighed and placed in each cage 90 minutes post-injection. HFD was measured at 2 hours after placement into the food hopper and again 24 hours later. JZL195 was dissolved in a vehicle comprised of 90% saline, 5% Cremophor, 3.4% ethanol and 1.6% DMSO.

### Data Analysis

Food intake, body weight, and blood glucose data were analyzed using two-way ANOVA (genotype x time or genotype x dose) with Bonferroni post-hoc tests. For experiments involving JZL195 treatment, food intake data were analyzed using two-way ANOVA with repeated measures. Area under the curve values (AUC) were calculated for blood glucose levels during glucose and insulin tolerance tests, and for JZL195 treatment. All statistics testing was done using GraphPad Prism 6 (GraphPad Software, La Jolla, CA). Values are expressed as ± SEM.

## Results

### Food Intake and Body Weight

**Fig 1:**
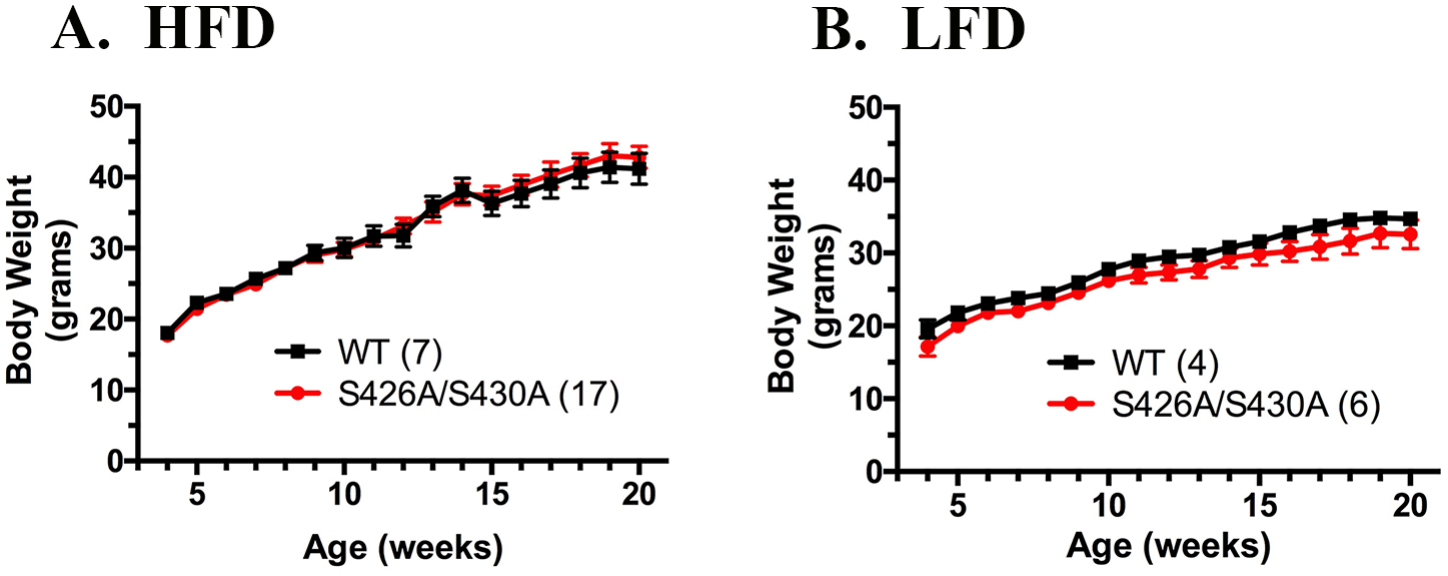
S426A/S430A mutant mice have normal body weight. Body weight was measured each week for S426A/S430A mutant (red line and circles) and wild-type mice (WT; black line and squares) given HFD (**A**) or LFD (**B**) starting from weaning until 20 weeks of age. Data are expressed as mean ± S.E.M and were analyzed using two-way ANOVA. The number of animals tested in each group is indicated in parentheses.

**Fig 2:**
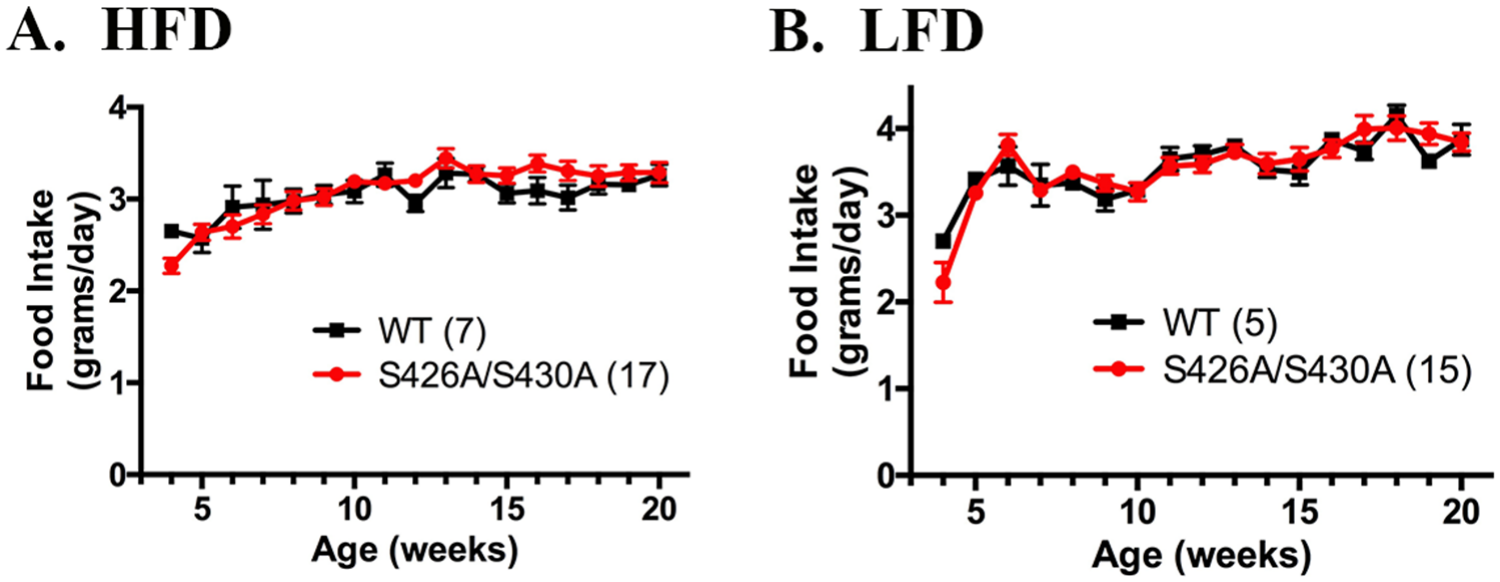
S426A/S430A mutant and wild-type mice consume equal amounts of food. Average daily food consumption was assessed for each week in S426A/S430A mutant mice (red line and circles) and wild-type (WT; black line and squares) mice given either HFD (**A**) or LFD (**B**) starting from weaning until 20 weeks of age. Data are expressed as mean ± S.E.M and were analyzed using two-way ANOVA. The number of animals tested in each group is indicated in parentheses.

Daily food intake and weekly body weight were measured in S426A/S430A mutant and wild type mice from weaning until 20 weeks of age. S426A/S430A mutant and wild-type mice given HFD displayed nearly identical body weights throughout the course of this experiment (Fig 1A). Average body weight of S426A/S430A mutant mice given control LFD also did not differ from diet-matched wild-type controls at any age (Fig 1B). Daily food intake was calculated and plotted as average daily food consumption in grams per day for each week. Mice expressing the S426A/S430A mutation did not exhibit any difference in consumption of either HFD or control LFD at any age (Fig 2A and 2B). Thus we saw no evidence of abnormal body weight regulation or feeding behavior in S426A/S430A mutant mice compared to wild-type littermate controls. Similar results were found in male and female mice.

### Glucose Homeostasis

**Fig 3:**
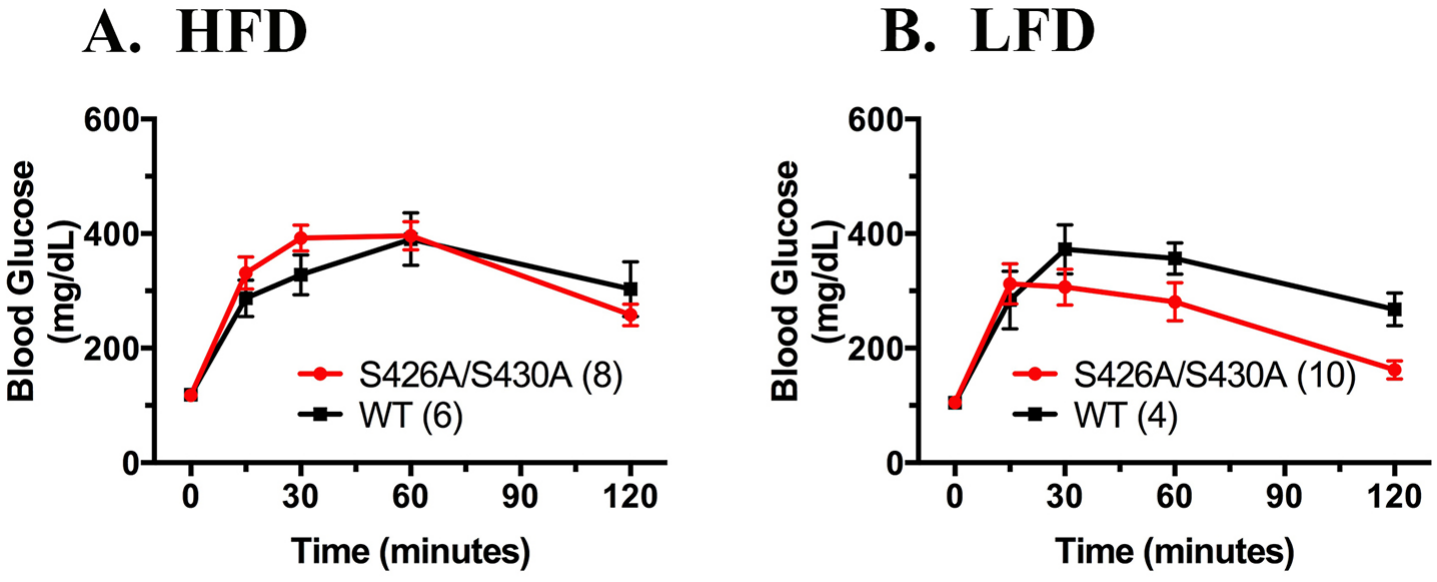
S426A/S430A mutant mice given LFD but not HFD exhibit greater glucose tolerance compared to wild-type controls. Fasting blood glucose (t = 0) and blood glucose levels at 15, 30, 60, and 120 minutes following intraperitoneal administration of 2g/kg glucose were measured in 20 week old S426A/S430A mutant (red circles and line) and wild-type (WT) littermates (black squares and line) fed either HFD (**A**) or LFD (**B**). Data are expressed as mean ± S.E.M and were analyzed using two-way ANOVA. The number of animals tested in each group is indicated in parentheses.

Glucose homeostasis was also examined in 20 week old S426A/S430A mutant and wild-type mice given either HFD or LFD diet starting at weaning. Fasting blood glucose levels were measured after an overnight 18 hour fast in S426A/S430A mutants and were not significantly different than fasted wild-type control mice (Fig 3). Glucose tolerance was measured in S426A/S430A mutant and wild-type mice given an IP injection of 2 g/kg D-glucose. Glucose tolerance was not significantly different between HFD-fed S426A/S430A mutant and wild-type mice (Fig 3A). Surprisingly, S426A/S430A given LFD displayed a slight improvement in glucose tolerance relative to diet-matched wild-type mice (F_1, 60_ = 4.265; *P* = 0.043; Fig 3B). However, area under the curve (AUC) analysis failed to find any significant differences between the genotypes (S426A/S430A, 29,821 ± 2634; WT, 37,500 ± 2477, p = 0.12).

### Fed-Fast Re-Feeding

**Fig 4:**
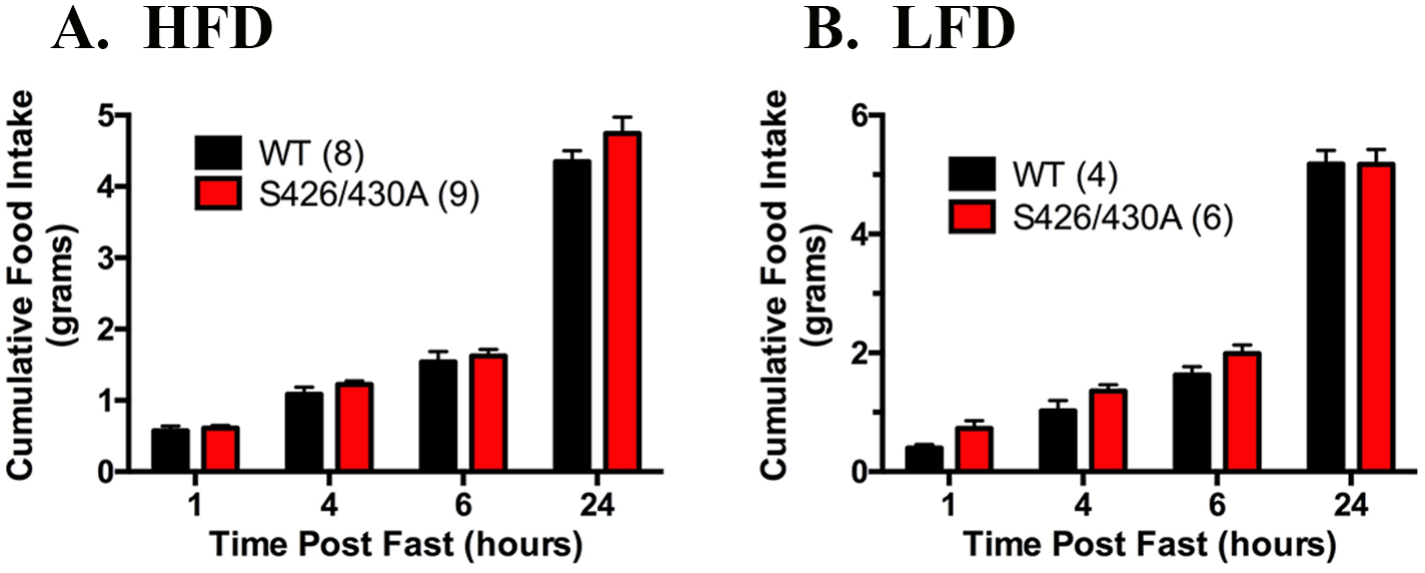
S426A/S430A mutants exhibit normal re-feeding following an overnight fast. Re-feeding was assessed in 20 week old S426A/S430A mutant (red bars) and wild-type (WT; black bars) mice given HFD (**A**) or LFD (**B**) from weaning to 20 weeks of age. Consumption of HFD or LFD was measured in fasted mice at 1, 4, 6, and 24 hours following restoration of food access. Data are expressed as mean ± S.E.M and were analyzed using two-way ANOVA. The number of animals tested in each group is indicated in parentheses.

Re-feeding after an overnight fast was examined to assess feeding in a nutrient-deficient state. Consumption of HFD or LFD in S426A/S430A mutant and wild type mice was assessed 1, 4, 6, and 24 hours after the restoration of food access following an overnight fast. Consumption of HFD during re-feeding was not changed in S426A/S430A mutants compared to wild-type control mice for any time point (Fig 4A). Similarly, post-fast re-feeding values for LFD control diet in S426A/S430A mutant and wild-type mice were also not significantly different at any time point (Fig 4B). However, S426A/S430A mutant mice trended to eat more than wildtypes at 1 hour, and this value approached significance (p = 0.10, unpaired t-test). Overall, these data mirror the results of experiments examining ad libitum HFD and LFD consumption under non-fasting conditions (Fig 2) and suggest that HFD and LFD consumption is not altered in S426A/S430A mutant mice, even after fasting.

### Insulin Tolerance

**Fig 5:**
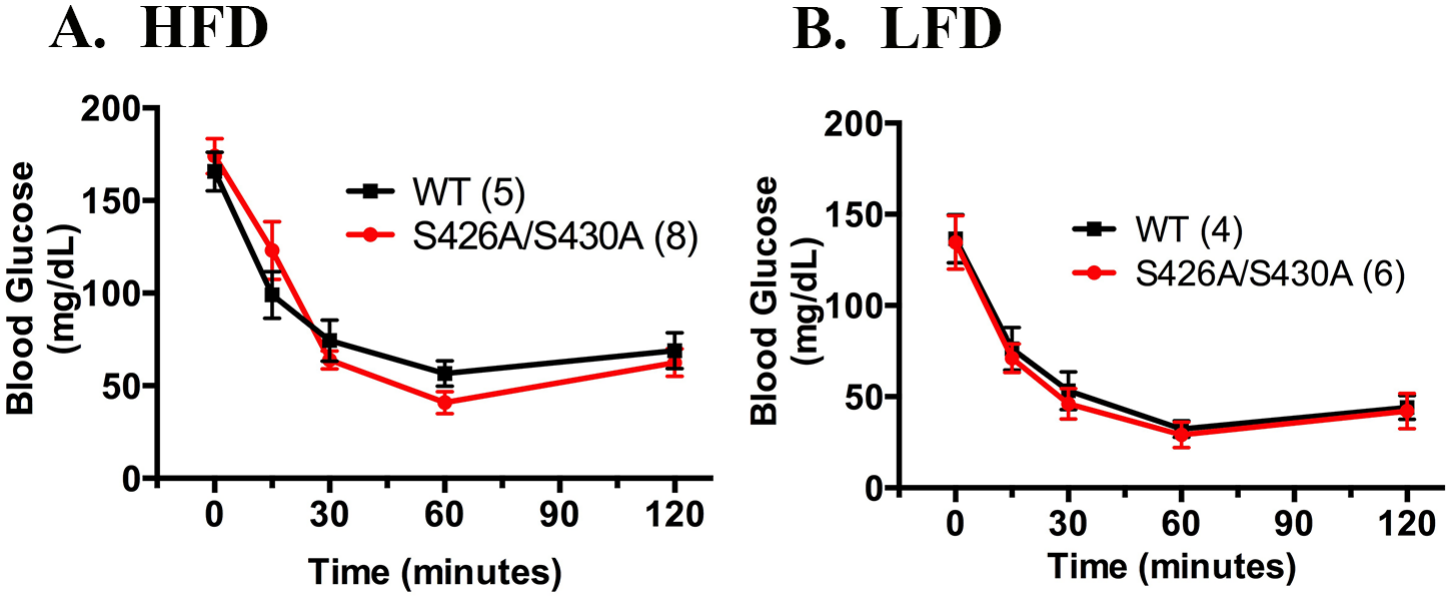
S426A/S430A mutants possess normal insulin sensitivity. Fasting blood glucose (t = 0) and blood glucose levels at 15, 30, 60, and 120 minutes after intraperitoneal administration of 1 U/kg insulin were measured in HFD (**A**) or LFD (**B**) fed S426A/S430A mutant (red circles and line) and wild-type (WT) littermates (black squares and line) at 30 weeks of age. Data are expressed as mean ± S.E.M and were analyzed using two-way ANOVA. The number of animals tested in each group is indicated in parentheses.

Sensitivity to 1 U/kg insulin was examined in HFD and LFD-fed S426A/S430A mutant and wild-type mice fasted overnight for 18 hours. Insulin tolerance testing was conducted at 30 weeks of age in S426A/S430A mutant and wild-type mice given either HFD or LFD control diet starting immediately after weaning. Insulin sensitivity was assessed by measuring the decrease in blood glucose levels occurring after treatment with 1 U/kg insulin. S426A/S430A mutant and wild-type mice exhibit pronounced decreases in blood glucose levels following administration of 1 U/kg Insulin. However, there were no differences in insulin sensitivity at any time point between S426A/S430A mutant and wild-type mice subjected to either HFD (Fig 5A) or LFD (Fig 5B).

### Δ^9^-THC-induced Feeding Dose Response Curve

**Fig 6:**
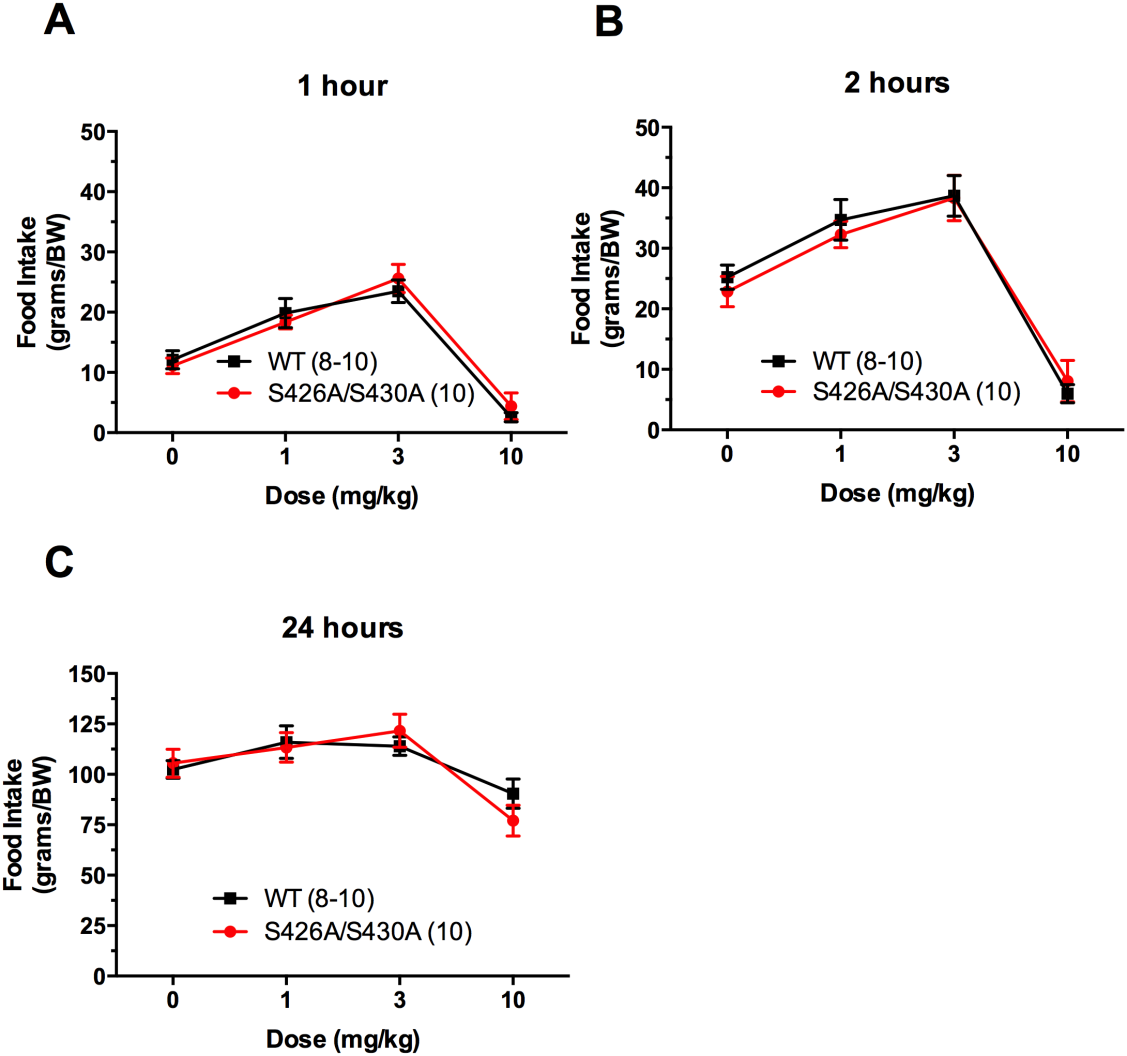
S426A/S430A mutants have similar sensitivity to Δ^9^-THC-induced feeding compared to wildtype control mice. Individual food intake values were measured 1, 2 and 24 hours after a 30 minute pretreatment with 0, 1, 3, or 10 mg/kg Δ^9^-THC. Δ^9^-THC increased food intake in S426A/S430A mutant (red circles and line) and wild-type (WT) littermates (black squares and line) 1 hour (A) and 2 hours (B) after injection. 24 hour food intake is shown in (C). 10 mg/kg Δ^9^-THC suppressed food intake similarly in both genotypes at all time points (A-C). Mice were fasted for 1 hour prior to intraperitoneal injections of Δ^9^-THC. Food intakes are expressed as grams of food per kilogram of body weight (BW). Data are expressed as mean ± S.E.M and were analyzed using two-way ANOVA. The number of animals tested in each group is indicated in parentheses.

In order to determine if S426A/S430A mutant mice are more sensitive to the ability of Δ^9^-THC to stimulate food-intake, dose-response curves were generated for S426A/S430A mutant and wildtype mice. Food intake was measured 1, 2, and 24 hours after an injection of Δ^9^-THC. 1 and 3 mg/kg Δ^9^-THC stimulated food intake at both 1 hour (F_3, 68_ = 53.57, p<0.0001) and 2 hours (F_3, 68_ = 46.48, p<0.0001) after injection (Fig 6A and 6B). 10 mg/kg caused significant hypolocomotion and thus food intake was minimal 1 and 2 hours after this dose. At 24 hours, there was a persistent reduction in food intake in mice injected with 10 mg/kg Δ^9^-THC (Fig 6C; F_3, 68_ = 9.904; P<0.0001). No differences were observed between intakes of S426A/S430A mutant and wildtype mice indicating that both genotypes responded similarly to Δ^9^-THC-induced food intake. All mice were fasted for 1 hour prior, injected with 0, 1, 3, and 10 mg/kg Δ^9^-THC, and presented with food 30 minutes later. Of note, the ability 1 and 3 mg/kg Δ^9^-THC to stimulate food intake was not observed unless mice were fasted prior to Δ^9^-THC treatment. Body weights were measured prior to each injection and they remained unchanged throughout the study.

### JZL195-induced Feeding

**Fig 7:**
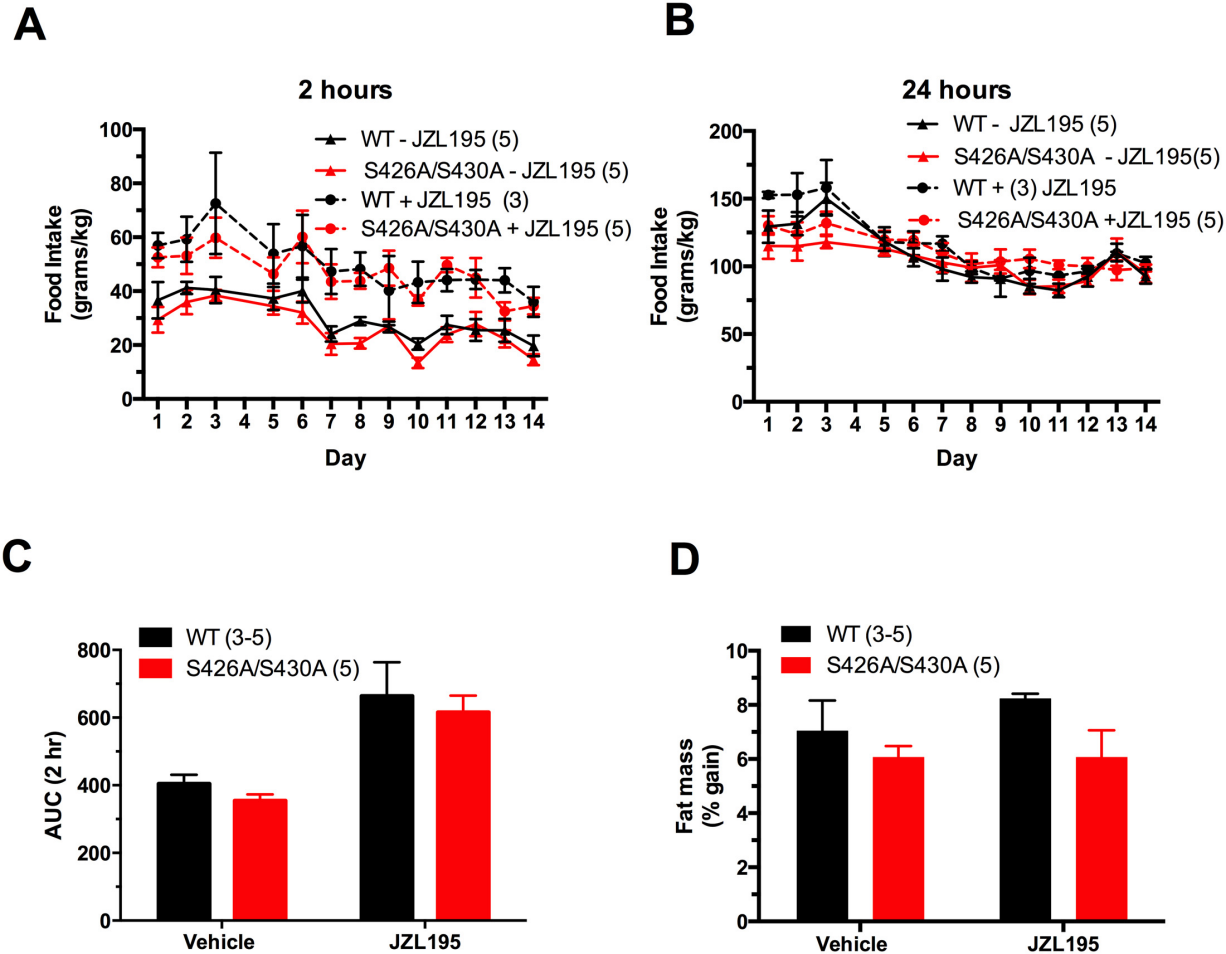
Repeated injections of JZL195 increases 2 hour food intake similarly in S426A/S430A mutant and wildtype control mice. JZL195 was administered daily for 14 consecutive days at a dose of 8 mg/kg. Food intake was increased 2 hours after a 90 minute pretreatment with JZL195 in S426A/S430A mutant (KI+; red circles and dotted line) and wild-type littermates (WT+; black circles and dotted line) (A), but not after 24 hours (B). Responses from vehicle treated control mice are also shown in A and B (KI-; red triangles and solid line and WT-; black triangles and solid line). Day 4 data was omitted from analysis in A and B. Food intakes are expressed as grams of food per kilogram of body weight (BW). Shown in C is the area under the curve (AUC) analysis for 2 hour food intake data from A. Shown in D are the values for the total percent of fat mass gained during the 14 day treatment period. Data are expressed as mean ± S.E.M and were analyzed using two-way ANOVA with repeated measures (A and B) or two-way ANOVA (C and D). The number of animals tested in each group is indicated in parentheses.

Fatty acid amide hydrolase (FAAH) and monoacylglycerol lipase (MAGL) catalyze the breakdown of AEA and 2-AG, respectively. JZL195 is a dual FAAH/MAGL inhibitor that has previously be shown to raise endocannabinoid levels, but has yet to be tested for its effect on food intake in mice [25]. Repeated daily injections of 8 mg/kg JZL195 significantly increased 2 hour food intake over the 14 day treatment period (Fig 7A) (F_3, 14_ = 11.89; P = 0.0004), but did not increase 24 hour food intake levels in either S426A/S430A mutant or wildtype mice (Fig 7B). No differences in food intake were found between the two genotypes at any timepoint. However, an overall decline in both 2 hour and 24 hour food intakes were observed from day 1 to day 14. Similar results were obtained with area under the curve analysis where there was a significant effect of JZL195 (F_1, 14_ = 31.24; P<0.0001), but no effect of genotype (Fig 7C). Analysis of body composition using TD-NMR showed no differences in the initial percent fat mass of S426A/S430A mutant and wildtype mice. Furthermore, no change in percent fat mass occurred between the genotypes after 14 days of 8 mg/kg JZL195 treatment (Fig 7D).

## Discussion

The endocannabinoid system has been shown to be an important regulator of feeding control and metabolic function. Anecdotally, the ability of recreationally consumed cannabis to stimulate intake of palatable food in humans has been widely reported. In pre-clinical models, treatment with endocannabinoids such as AEA or exogenous cannabinoids such as Δ^9^-THC can stimulate feeding behavior [13,26–30]. Synthetic Δ^9^-THC, known commercially as Dronabinol, has been approved for the treatment of cachexia and anorexia in AIDS and cancer patients by the United States Food and Drug Administration [31–33].

Conversely, the CB_1_-selective inverse agonist, SR141716 (Rimonabant) has been shown to improve multiple parameters associated with normal metabolic physiology. In rodent models these beneficial effects include reduced consumption of palatable food, improved glucose homeostasis, increased insulin sensitivity, increased energy expenditure, and improved levels of circulating lipids [7–11,34–36]. These results with Rimonabant are supported by CB_1_ knock out (KO) mice which display decreased body mass and adiposity, decreased food intake, and resistance to diet-induced obesity [4,5]. In clinical trials, Rimonabant was found to decrease body weight and waist circumference, and reduced circulating glucose, leptin, and triglycerides in obese humans [37].

Further work has demonstrated that circulating AEA and 2-AG are increased in obese humans suggesting the possibility that the peripheral endocannabinoid system is activated in obesity [38]. Work has shown that increasing endocannabinoid levels in mice leads to increased levels of circulating triglycerides due to impaired apolipoprotein E-mediated lipid clearance [19]. However, relative to the wealth of accumulated research on the effects of pharmacological and genetic inactivation of CB_1_, much less is known about the effects of endocannabinoid system activation on metabolism. Therefore, we undertook this study to address this question using S426A/S430A mutant mice. These mice are more sensitive to endocannabinoids as well as exogenous plant-derived cannabinoids such as Δ^9^-THC [24]. To this end, we examined food intake under both fed and fasted conditions, body weight, glucose clearance following a glucose challenge, and insulin tolerance in S426A/S430A mutant mice.

Surprisingly, the metabolic phenotype of these mice was modest: S426A/S430A mutant mice given either HFD or control LFD displayed no differences in food consumption or body weight compared to wild-type littermate controls. Additionally, S426A/S430A mutant mice given either HFD or LFD exhibited similar insulin tolerance as diet and age-matched wild-type controls at 30 weeks of age. The only significant metabolic phenotype detected in S426A/S430A mutant mice was the observation that mutants given LFD unexpectedly display improved glucose tolerance compared to diet matched wild-type controls (Fig 3B). However, this difference in glucose clearance between S426A/S430A mutants and wild-type littermates did not extend to mutant mice given HFD.

Early during the phenotypic characterization of S426A/S430A mutant mice we hypothesized that the relative lack of metabolic phenotype might be due to down-regulation of CB_1_ and/or decreased amounts of endocannabinoids. This idea was based in part on the finding that mice lacking CB_1_ exhibit improved glucose homeostasis relative to wild-type mice. Blockade of CB_1_ receptors has been shown to improve insulin resistance and glucose clearance in obese rats while CB_1_ agonists have been found to induce glucose intolerance [39,40]. Therefore, we wanted to examine glucose tolerance in S426A/S430A mutant mice expressing a form of CB_1_ that is more sensitive to endocannabinoids and Δ^9^-THC. The finding that S426A/S430A mutant mice exhibit a similar improvement in glucose tolerance compared to wild-type mice suggested the possibility that the S426A/S430A mutation might result in compensatory hypoactive endocannabinoid signaling. Our recent work demonstrated normal levels of AEA and 2-AG throughout most regions of the brain and normal CB_1_ expression in the hippocampus, striatum, and forebrain of S426A/S430A mutant mice [24]. The number of CB_1_ [^3^H]CP55,940 binding sites (B_max_) was not different in S426A/S430A mutant mice compared to wild-type mice. Additionally, the E_max_ for CP55,940-stimulated [^35^S]GTPγS binding in the spinal cord was also similar in S426A/S430A mutant compared to wild-type mice demonstrating that the mutant form of CB_1_ can be activated by agonist to an equivalent extent [24]. However, lower levels of 2-AG in the cortex and CB_1_ in the cerebellum were detected in S426A/S430A mutant mice. This raises the possibility that S426A/S430A mutants might possess region-specific dysregulation of endocannabinoid signaling in brain regions responsible for metabolic regulation such as the hypothalamus. Alternatively, CB_1_ receptor desensitization might be less critical in some regions, a hypothesis that is supported by findings demonstrating that repeated Δ^9^-THC administration resulted in significant CB_1_ desensitization in the prefrontal cortex, amygdala and hippocampus but no CB_1_ desensitization was observed in the striatum [41]. Additional studies have also shown that the degree of tolerance to cannabinoids and CB_1_ desensitization is behavior and region specific [42–44]. A further study demonstrated that the degree of CB_1_ desensitization in response to agonist is sex and age-specific [45], raising the possibility that female S426A/S430A mutant mice might have a distinct metabolic phenotype if compared to males. Conditional S426A/S430A mutant mice might help in dissecting out any region specific effects of CB_1_ receptor hyperactivity on specific metabolic parameters.

Our previous work found no differences in baseline body temperature or tail-flick antinociception in untreated S426A/S430A mutants [24]. The increased cannabinoid response reported in S426A/S430A mutant mice was detected only when exogenous Δ^9^-THC was administered or endocannabinoid levels were increased using an inhibitor of endocannabinoid breakdown [24]. Therefore, it is possible that changes in metabolic homeostasis such as food intake or body weight might only occur in the presence of exogenous cannabinoid agonists in S426A/S430A mutants. In order to address this, we generated a dose-response curve for Δ^9^-THC-induced feeding. As expected, lower doses of Δ^9^-THC significantly and dose-dependently increased food intake 1 and 2 hours after its administration (Fig 6). However, there was no evidence that S426A/S430A mutant mice were more sensitive to an exogenously administered cannabinoid agonist in regards to its ability to acutely stimulate food intake. These data collectively suggest that the sensitivity of S426A/S430A mutant mice to the behavioral, metabolic and other physiological effects such as hypothermia to exogenously administered cannabinoid agonists are separable. These data also provide further evidence for region-specific dysregulation of endocannabinoid signaling in distinct brain regions as described above.

To further explore endocannabinoid-stimulated hyperphagia in S426A/S430A mutant mice, the dual inhibitor of FAAH and MAGL JZL195 was administered daily for two weeks. To our knowledge, this is the first study reporting the effects of JZL195 on food intake in mice. However, a prior study demonstrated that injection of the FAAH inhibitor AA5HT into the ventral striatum of rats stimulated 1 and 4 hour food intake [46]. In our mice, JZL195 significantly increased food intake 2 hours after injection suggesting that overall elevation of AEA and 2-AG endocannabinoid levels acutely stimulates food intake in mice. This dose of 8mg/kg JZL195 has previously been shown to raise AEA and 2-AG levels 4–6 times above baseline levels, respectively, [25] and thus supports the overarching hypothesis that elevated endocannabinoid levels promotes hyperphagia. However, similar to our findings with Δ^9^-THC, there were no differences in food intake between S426A/S430A mutant and wildtype control mice suggesting that at least for cannabinoid–induced hyperphagia S426A/S430A mutants were no more sensitive than wildtype controls to this dose of JZL195. Interestingly, we have previously shown that doses up to 10mg/kg caused very minimal analgesic and hypothermic responses in both wildtype and S426A/S430A mutant mice. However, S426A/S430A mutant mice were significantly more sensitive to both the analgesic and hypothermic effects of 30 mg/kg JZL195 [24]. Therefore, it is possible that S426A/S430A mutant mice would have increased food intake compared to wildtype controls if higher doses of JZL195 were given. Furthermore, this work provides additional evidence that the analgesic, hypothermic and food intake effects of cannabinoids are separable.

Previous studies have shown that peripherally-restricted CB_1_ antagonists are as effective as rimonabant in reducing body weight, reducing lipids and improving insulin sensitivity in rodents [47,48] that are due, in part, to direct effects on peripheral tissues including adipose, liver, skeletal muscle and pancreas [9,49]. A major limitation of our study is that we have not measured circulating levels of endocannabinoids or their levels in any of these peripheral tissues in S426A/S430A mutant mice. Moreover, CB_1_ expression levels and functionality have not been assessed nor has the levels of endocannabinoid synthetic and degradative enzymes. Thus, we cannot rule out that compensatory changes in peripheral tissues, including a decrease in circulating endocannabinoid levels, might be attenuating the obesogenic effects that would be expected in our mutant mice.

To summarize, our previous phenotypic characterization of S426A/S430A mutant mice revealed an exaggerated and prolonged response to both exogenous and endogenous cannabinoids [24]. Due to the extensive literature surrounding the involvement of the endocannabinoid system in metabolic control we anticipated that these mutant mice would display metabolic abnormalities such as increased food intake and body weight and reduced glucose tolerance and insulin sensitivity. However, we do not observe evidence of metabolic abnormalities in S426A/S430A mutant compared to wild-type controls with the exception of modestly improved glucose tolerance in mutants given LFD. One possible explanation for the lack of metabolic phenotype could be that the S426A/S430A mutation isn’t sufficient to confer significantly enhanced responses to relatively low basal levels of endocannabinoids in tissues or circulation. Furthermore, the S426A/S430A mutation does not confer additional sensitivity to the hyperphagia promoted by pharmacologically-induced increases in endocannabinoid signaling pathways. Therefore, we conclude that this model is likely to have limited utility for studying endocannabinoid system roles in metabolic regulation.

## Supporting Information

S1 File Fig 1A Data Files. (PZFX)

S2 File Fig 1B Data Files. (PZF)

S3 File Fig 2A Data Files. (PZF)

S4 File Fig 2B Data Files. (PZF)

S5 File Fig 3A Data Files. (PZFX)

S6 File Fig 3B Data Files. (PZFX)

S7 File Fig 4A Data Files. (PZF)

S8 File Fig 4B Data Files. (PZF)

S9 File Fig 5A Data Files. (PZFX)

S10 File Fig 5B Data Files. (PZF)

S11 File Fig 6 Data Files. (PZFX)

S12 File Fig 7 Data Files. (PZFX)
